# A Swedish randomized controlled trial of early identification and brief intervention for alcohol use disorders: preliminary findings

**DOI:** 10.1186/1940-0640-7-S1-A56

**Published:** 2012-10-09

**Authors:** Hanna Reinholdz, Fredrik Spak, Agneta Ronstad

**Affiliations:** 1 Department of Social Medicine, Gothenburg University, Gothenburg, Sweden

## 

In many countries, as in Sweden, it has been difficult to integrate early identification and brief intervention (EIBI) into daily primary health care (PHC) practice. We describe an ongoing study (the Secondary Prevention, Implementation, and Research on Alcohol [SPIRA] study) investigating possible solutions to overcome implementation obstacles and methods that facilitate the implementation process. This is the first Swedish EIBI study to include cost-effectiveness. The PHC practices have been cluster-randomized into four groups to test implementation strategies. It is estimated that 800-1000 patients will be recruited and will receive EIBI in two four-week periods, six months apart. The total project time is 14 months from baseline to follow-up, which will be done by telephone six months after the last intervention period. Two models are being tested: 1) the detection of risky drinking with screening (Alcohol Use Disorders Identification Test-Consumption [AUDIT-C]) versus other methods of EI, and 2) whether an implementation coach increases the number of BI conducted by PHC providers beyond what an education package does. The implementation process is followed by focus group interviews. The SPIRA study is ongoing. So far, 15 PHC practices are involved in different counties of Sweden. Three have participated in two intervention periods, the rest in one. Twenty-three focus groups have been conducted. According to preliminary findings, many PHC practices wish to be involved in lifestyle interventions as they become mandatory; but, at the same time, many general practitioners and nurses are reluctant to work on what they do not consider to be a main treatment task. There is a considerable uncertainty as to what risky drinking is, particularly when alcohol problems have not yet appeared or been disclosed. Implementation coaches that assist with EIBI are much requested by PHC providers, indicating that such support will be necessary if EIBI is to be adopted and delivered on a large scale in Sweden.

